# SensA: web-based sensitivity analysis of SBML models

**DOI:** 10.1093/bioinformatics/btu378

**Published:** 2014-06-05

**Authors:** Max Floettmann, Jannis Uhlendorf, Till Scharp, Edda Klipp, Thomas W. Spiesser

**Affiliations:** Theoretical Biophysics, Humboldt-Universität zu Berlin, Invalidenstr. 42, 10115 Berlin, Germany

## Abstract

**Summary:** SensA is a web-based application for sensitivity analysis of mathematical models. The sensitivity analysis is based on metabolic control analysis, computing the local, global and time-dependent properties of model components. Interactive visualization facilitates interpretation of usually complex results. SensA can contribute to the analysis, adjustment and understanding of mathematical models for dynamic systems.

**Availability and implementation:** SensA is available at http://gofid.biologie.hu-berlin.de/ and can be used with any modern browser. The source code can be found at https://bitbucket.org/floettma/sensa/ (MIT license)

**Contact:** max.floettmann@biologie.hu-berlin.de or thomas.spiesser@biologie.hu-berlin.de

## 1 INTRODUCTION

The understanding of complex systems and their dynamics has greatly improved with mathematical modelling. With models, the dynamics of system components can be analysed, hypotheses can be tested and the behaviour of the system can be predicted in different conditions or in response to perturbations. These predictions guide future experiments, which can save money and time.

To mimic the behaviour of the real biological system, model parameters have to be tuned based on biological observations. In this process, it is vital to test the effects of changes in parameter values on the behaviour of the system. This test is often referred to as sensitivity analysis. Sensitivity analysis measures the change of a specific system property (e.g. a steady state concentration, reaction flux or the amplitude of oscillations) in response to changes in parameter values. Thus, it shows how sensitive the system is towards a particular parameter. It can also be interpreted as fragility or robustness analysis of the system.

Here, we implement sensitivity analysis as defined by metabolic control analysis (MCA). MCA defines coefficients that describe the effect of infinitesimal changes of parameters on system properties, like reaction fluxes or variable concentrations (Heinrich and Rapoport, 1974; Kacser and Burns, 1973). Classical MCA is limited to models in steady state, but Ingalls and Sauro extended the theory to look at the time-dependent changes of sensitivities as well (Ingalls and Sauro, 2003). MCA and its extension provide a sound theoretical framework for sensitivity analysis.

SensA is a software to compute local, global and time-dependent sensitivity coefficients in models implemented in the Systems Biology Markup Language (SBML) (Hucka *et al.*, 2003), providing information about three distinct levels of sensitivities.

First, to analyse the influence of a parameter (or substrate) on an isolated reaction rate, we calculate local sensitivity coefficients (or elasticities). Second, to assess the effect of a perturbation of a parameter or reaction rate on the steady state fluxes or concentrations, we compute global response and control coefficients. In contrast to elasticities, control and response coefficients take into account the structure of the reaction network. In contrast to local coefficients, they can be useful to understand the impact of individual reactions or components on the behaviour of the system as a whole.

However, in case a dynamic system shows a transient response or oscillations (e.g. signalling cascades or the cell cycle), the influence of parameters may change over time. It can be of particular interest to analyse at what time a system is most sensitive, for example in cancer chronotherapy (Lévi *et al.*, 2010). Third, to provide an option to capture such effects, we compute time-dependent concentration response coefficients (TDCRCs) (Ingalls and Sauro, 2003). They allow to trace how sensitivities change over time.

Here, we present SensA, an online tool for sensitivity analysis. SensA is completely web-based, and requires no installation at all. A clear focus on sensitivities and a modern Javascript-based front-end make the tool easy to use. It extends sensitivity analysis as compared with standard modelling software [e.g. Copasi (Hoops *et al.*, 2006) or JWS online (Olivier and Snoep, 2004)] to include time-dependent sensitivities as well. Thus, SensA offers the most complete set of sensitivity analysis we know of.

## 2 IMPLEMENTATION

The calculations are implemented in Python and are available as open source software (see Availability and implementation). We provide details on the numerical computation of the sensitivities on the project’s website.

The front-end is implemented in meteor.js and can be accessed using a standard compliant browser. Users can upload models in SBML format, and every model is analysed in its own process on our server. Thus, several models can be analysed at the same time.

**Fig. 1.**
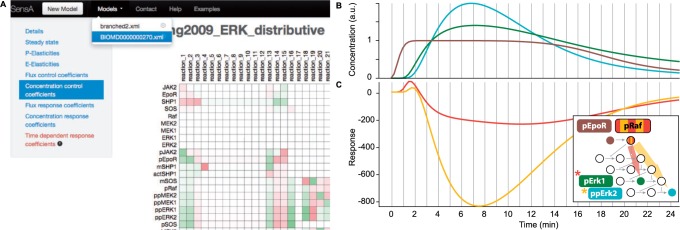
(**A**) Screenshot of the SensA user interface displaying concentration control coefficients in a matrix for the ERK cascade model from Schilling *et al.* (2009). (**B**) Time course simulation of concentrations of pEpoR, pErk1 and ppErk2. (**C**) Time-dependent response coefficients of pErk1 and ppErk2 with respect to changes in pRaf over time, as calculated by our software

On upload of a model, the analysis starts and the different sensitivities are displayed with reactive vector-graphics (.svg) as soon as they become available (Fig. 1A). A user can already investigate some results while more time-consuming calculations may still be running. Additionally, we provide a model overview and an option to simulate the time courses of model variables to enable users to check that the model works correctly. Graphics and data (.csv) can be downloaded for further processing.

All uploaded models and generated data can be deleted by the user. Also, the analysis software is usable as command-line tool on a local computer through its command-line user interface.

## 3 DISCUSSION

To demonstrate the main analysis and the corresponding type of results a user can expect, we analysed a model for the extracellular signal-regulated kinase (ERK) cascade from Schilling *et al.* (2009), accessible on the Biomodels database (BioModels ID: BIOMD0000000270). The model comprises 33 variables and 39 parameters, resulting in 2376 different TDCRCs. A schematic of the model topology and a selection of concentration time courses and computed TDCRCs are shown in Figure 1B. Looking at the structure of the model and the concentrations, it becomes clear that a phosphorylation of pRaf leads to a number of phosphorylations further downstream. Using SensA, we are now able to observe the inherent relationship between changes in the concentration of pRaf and pErk1 and ppErk2 over time.

Moderately complex models already produce a large number of TDCRCs that can be problematic to visualize. To address this, we implemented interactive graphics with a selection matrix and a plotting area. The matrix shows all possible TDCRCs. When the user hovers over a specific coefficient, the line is transiently displayed in the plot. This serves as a quick and easy way to scan a large number of coefficients. Also, the user may select to plot all, none or the 10 most extreme coefficients.

## 4 CONCLUSION

Sensitivity analysis in general is an important tool in many areas of modern systems biology and is often used to understand the growing complexity of models. Especially TDCRCs can give an interesting perspective on signalling models, and are an often cited method in the field (original paper has ∼140 citations). Nevertheless, studies that actually use it are rare (Petelenz-Kurdziel *et al.*, 2013). We provide SensA to close the gap between this sophisticated analysis and a comprehensive way to use it. This can enable modellers to use the method and make the results more accessible.

*Funding*: This work was supported by BMBF (ViroSign - 0316180A; Translucent - 0315786A) to E.K. and by the Deutsche Forschungsgemeinschaft (GRK 1772 CSB).

*Conflict of Interest*: none declared.

## References

[btu378-B1] Heinrich R, Rapoport TA (1974). A linear steady-state treatment of enzymatic chains. General properties, control and effector strength. Eur. J. Biochem.

[btu378-B2] Hoops S (2006). COPASI–a COmplex PAthway SImulator. Bioinformatics.

[btu378-B3] Hucka M (2003). The systems biology markup language (SBML): a medium for representation and exchange of biochemical network models. Bioinformatics.

[btu378-B4] Ingalls BP, Sauro HM (2003). Sensitivity analysis of stoichiometric networks: an extension of metabolic control analysis to non-steady state trajectories. J. Theor. Biol..

[btu378-B5] Kacser H, Burns JA (1973). The control of flux. Symp. Soc. Exp. Biol..

[btu378-B6] Lévi F (2010). Circadian timing in cancer treatments. Annu. Rev. Pharmacol. Toxicol..

[btu378-B7] Olivier BG, Snoep JL (2004). Web-based kinetic modelling using JWS online. Bioinformatics.

[btu378-B8] Petelenz-Kurdziel E (2013). Quantitative analysis of glycerol accumulation, glycolysis and growth under hyper osmotic stress. PLoS Comput. Biol..

[btu378-B9] Schilling M (2009). Theoretical and experimental analysis links isoform-specific ERK signalling to cell fate decisions. Mol. Syst. Biol..

